# Analysis of the effect of flow channel structures on the centrifugal accelerated motion of particles in a vacuum state

**DOI:** 10.1038/s41598-024-59254-6

**Published:** 2024-04-15

**Authors:** Bo Sun, Shizhong Wei, Peng Li, Han Guo

**Affiliations:** 1 School of Mechatronics Engineering, Henan University of Science and Technology, Luoyang, 471003 Henan China; 2 School of Materials Science and Engineering, Henan University of Science and Technology, Luoyang, 471003 Henan China; 3 College of Agricultural Equipment Engineering, Henan University of Science and Technology, Luoyang, 471003 Henan China; 4 Office of Science and Technology, Henan University of Science and Technology, Luoyang, 471003 Henan China; 5 College of Mechanical and Electrical Engineering, Zhengzhou University of Light Industry, Zhengzhou, 450002 Henan China

**Keywords:** Particle, Centrifugal acceleration, Runner structure, Discrete-element method, Vacuum, Engineering, Mechanical engineering

## Abstract

In this study, the impact of flow channel structures on the acceleration of metal particles in a vacuum environment is explored, with the aim of enhancinge the acceleration quality in the centrifugal impact molding of metal powders. To assess this phenomenon, three evaluation indices are introduced: the average speed of particles thrown ${V}_{p}$, the average speed of the particles ${V}_{all}$, and the particle velocity distribution *V*_*f*_ (t). Additionally, the effects of six distinct runner structures on the centrifugal acceleration of the particles are analyzed in this research. The findings indicate that the arc-shaped flow channel structure not only ensures a more consistent acceleration process but also results in a higher ejection speed, leading to an improved acceleration effect. The unique contribution of this study is the examination of the relationship between flow channel designs and particle accelerations in a vacuum.

## Introduction

Particles are generally defined as tiny natural objects of a certain size and shape. Particles are typically classified into solid particles (e.g., powders), liquid particles (e.g., droplets and oil beads), and gas particles (e.g., bubbles in liquids)^1^. Numerous methods, including the airflow acceleration method^2^, mechanical acceleration method^3^, electromagnetic acceleration method^4^, ultrasonic acceleration method^5^, and thermal expansion acceleration method^6^ have been developed to accelerate particles to a certain speed, enabling various tasks such as separation, throwing, and impact crushing. Notably, that these methods are widely used in diverse fields such as intelligent manufacturing, chemical industry, weaponry, and mining^7–16^. In the context of centrifugal acceleration, the internal structure of different acceleration discs plays a crucial role in shaping the acceleration process. In this regard, Tan ^17^ conducted numerical simulations to optimize the design of the accelerating disc in a bipolar piston pusher and designed an accelerating disc to enhance the instantaneous velocity of particles by changing the disc structure and the blade geometry. G. Bell ^18^ conducted experimental and computational assessments of six feed accelerator designs utilized in decanter centrifuges. Additionally, a parametric study of drum and disc accelerators was carried out using computational models. Wang ^19^ developed a virtual prototype model of a vertical shaft impact crusher based on EDEM. Moreover, they simulated the motion stages and mechanical characteristics of material flow in rotors with different numbers of channels. The results indicated that the rotor with six channels exhibited the most reasonable structure, offering optimal acceleration effects and crushing efficiency. Feng ^20^ utilized discrete element simulation software to dynamically simulate the rotor, and analyze the particle velocities generated by the rotor. They focused on understanding the influence of the guide plate angle on the effects of particle acceleration. Guo ^21^ investigated the acceleration capacity of different channel models, considering particles of the same size. The analysis included an assessment of ejection velocities and ejection forces, revealing that the channel length and starting position significantly influenced the particle acceleration levels. Sun ^22^ developed a regression model that correlated runner modeling parameters with the particle ejection velocity. This model was developed through multifactor discrete element simulation experiments. The model was then utilized to optimize the design of internal runner parameters within the rotor and increase the particle ejection velocity. Liu ^23^ designed a conical disc with gradually decreasing (GD) curvature from top to bottom and derived governing equations based on theoretical analyses. The performance of the designed device was evaluated by considering various factors, such as the consistency of fertilizer applications and stability. Finally, the optimal busbar tilt angle was determined through the analysis. Shi ^24^ analyzed the relationship between fertilizer particle distributions and fertilizer thrower operating parameters using discrete element simulation experiments. They demonstrated a strong correlation between the turntable height of the fertilizer applicator and the coefficient of variation of fertilizer thrower distributions.

In this paper, the centrifugal acceleration rotor is taken as the research object. Without considering the disturbances of airflows on the particle motions and with reference to the relevant research on the centrifugal acceleration process of ores, seeds, feed and other particles, the discrete element simulation software EDEM is used to simulate the centrifugal acceleration process of particles inside the acceleration rotor. Additionally, the influence of different flow channel structures on the centrifugal acceleration process of particles is analyzed to guide the optimal design of the centrifugal acceleration rotor structure to improve the centrifugal acceleration efficiency of powder particles and provide support for the realization of jet impact molding.

## Materials and methods

### Centrifugal motion of the particles

**Figure 1:**
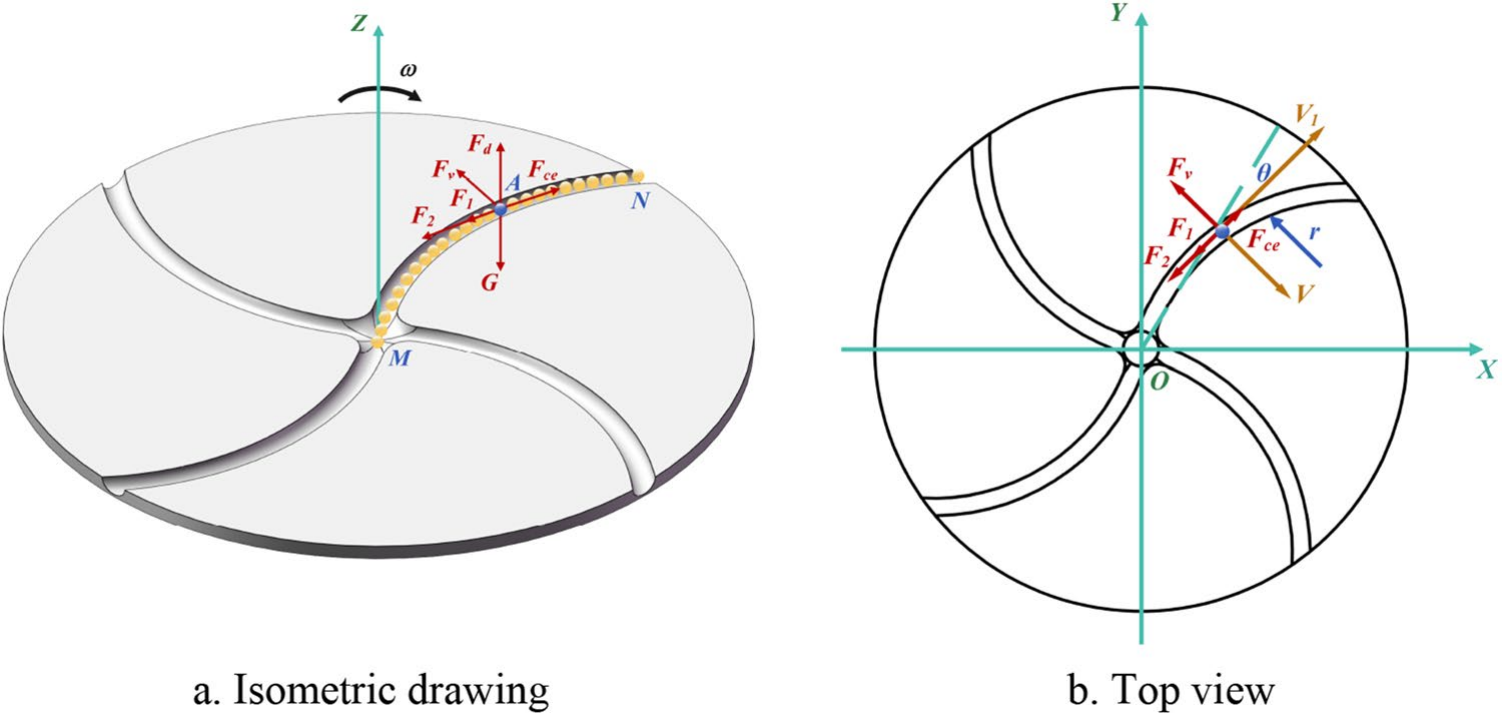
Force analysis of a particle on a centrifugal accelerator disk.

Granular media, characterized by macroscopic amorphous structures composed of mesoscopic discrete particles, exhibit various behavioral characteristics influenced by volume fractions and loading conditions. These behaviors include solid-like, liquid-like, gas-like, and inertial motion behaviors that are relatively unconstrained by neighboring particles ^25^. As the particle volume fraction decreases, the assumption of two-body collisions between particles is no longer satisfied, inter-particle collisions become improbable, and the external forces imposed on the particles dominate. At this point, the particles no longer adhere to the continuum mechanics principles governing liquid-like media. Instead, they enter an inertial state, following the laws of dynamics and motion for mass points. In this study, particles are considered rigid mass points with mass *m*, inter-particle interactions are ignored, and the impact of air resistance in a vacuum environment is not considered. When particles enter a centrifugal accelerating disc, particularly one with a circular arc runner structure, they rotate with the disc and accelerate along the channel MN until they exit. During this motion within the disc, the particles are subjected to various forces, including their own gravity *G*, the force on the bottom surface of the disc *F*_d_, the friction with the disc surface *F*_1_, the friction with the side of the flow channel *F*_2_, the centrifugal force *F*_ce_, and Koch's force *F*_v_. Figure 1 illustrates the force analysis of particles as they move to Point A.

The friction force acting on a particle on the bottom side of the disk can be expressed as follows:

1 $${F}_{1}=\mu mg$$

where *μ* is the coefficient of kinetic friction between the particles and the surface of the centrifugal disc and *m* is the mass of the particles, kg.

The centrifugal force applied to a particle is expressed as follows:

2 $${F}_{ce}=m\frac{{v}^{2}}{r}=m\frac{{\left(\omega r-\frac{dl}{dt}\mathit{sin}\theta \right)}^{2}}{r}$$

where $v$ is the tangential velocity of the particles with circular motion, m·s^−1^; $r$ is the radius of curvature of pPoint A, mm; $l$ is the length of the arc MA, mm; ${v}_{1}$ is the tangential velocity of the particles at Point A, m·s^−1^; $\theta$ is the angle between ${v}_{1}$ and the Line OA, rad; $\omega$ is the rotational angular velocity of the throwing disc, rad·s^−1^.

The Koch force can be obtained using the following expression:

3 $${F}_{co}=2m\omega \frac{dl}{dt}$$

Moreover, the frictional force acting on a particle on the lateral side of the disk is expressed as follows:

4 $${F}_{2}=\mu {F}_{co}=2\mu m\omega \frac{dl}{dt}$$

According to Newton's second law, the equation of motion for a particle moving along the MA direction is as follows:

5 $$m\frac{{d}^{2}l}{d{t}^{2}}=\frac{{\left(\omega r-\frac{dl}{dt}\mathit{sin}\theta \right)}^{2}}{r}m\mathit{cos}\theta -\mu mg-2\mu m\omega \frac{dl}{dt}$$

6 $$\frac{{d}^{2}l}{d{t}^{2}}=\frac{{\left(\omega r-\frac{dl}{dt}\mathit{sin}\theta \right)}^{2}}{r}\mathit{cos}\theta -\mu g-2\mu \omega \frac{dl}{dt}$$

where the parameters $l$, $r$, and $\theta$ are related to the structural characteristics of the rotor. Equation (6) indicates that the motion of particles in the accelerating rotor is influenced by the rotational speed and the structure of the turntable.

### Modeling and simulation

#### Centrifugal accelerator discs

**Figure 2:**
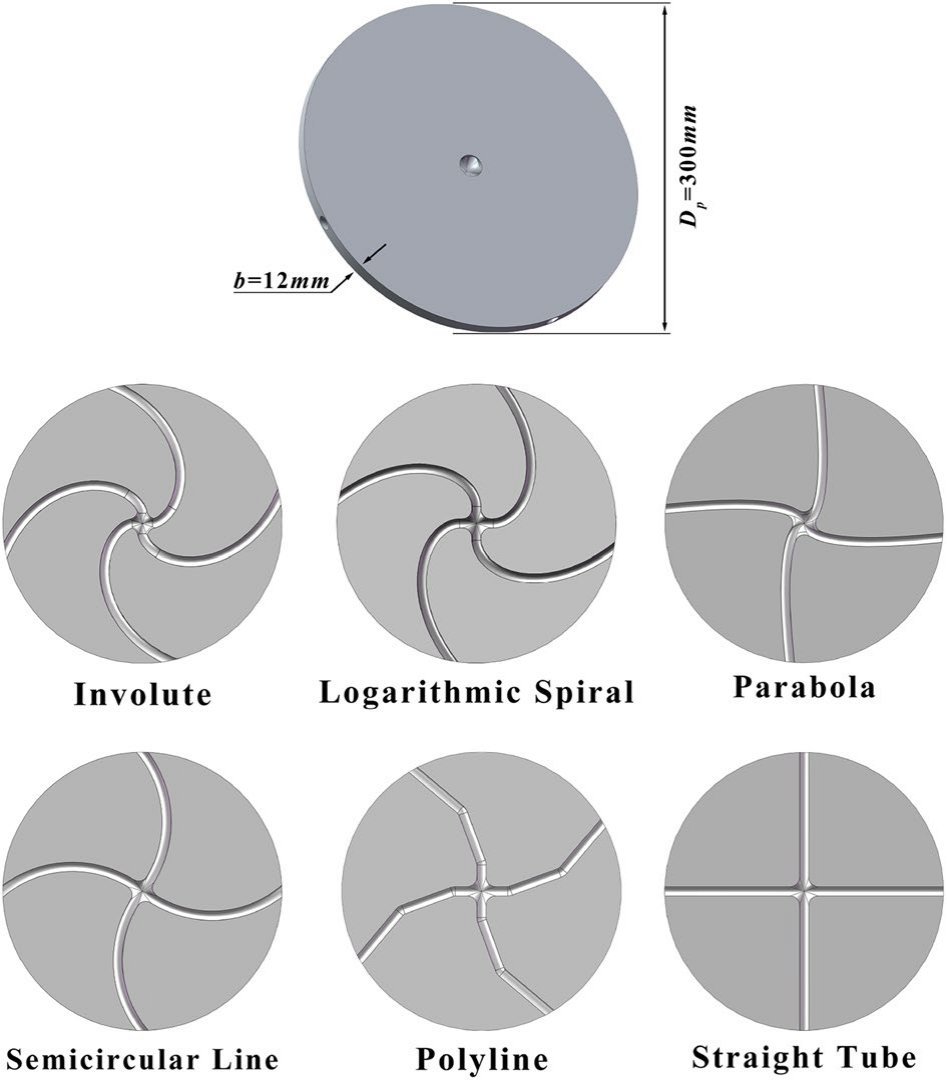
Accelerator disks with different runner structures.

Figure 2 illustrates centrifugal accelerating discs with six distinct runner structures: involute, logarithmic helix, parabolic, arc linear, straight-through, and multi-segment linear. The parameters of the accelerating disc, including the diameter (*D*_p_) and thickness (b), are constants. The disc body consists of the same material, structure, and material parameters as detailed in Tables 1 and 2.

**Table 1:** Structural parameters of the studied centrifugal acceleration turntable.

Parameters	Value
Outer diameter of the rotor ***D***_**p**_, mm	300
Thickness of the rotor ***b***, mm	12
Cross-sectional shape of the runner	circle
Cross-sectional dimensions ***d***, mm	10

**Table 2:** Properties of the rotor material.

Parameters	Value
Density of the material, kg∙m^3^	7.85 × 10^3^
Poisson's ratio of the material	0.3
Shear modulus of the material, MPa	2.06 × 10^3^

#### Particle

Regarding the physical characteristics necessary for particles to be used in forming techniques such as cold spraying, the focus of the present study is on metal powder particles characterized by a smooth surface, excellent fluidity, and predominantly spherical shape. Therefore, the particles are designated as spherical bulk, and the particles are simplified into a hard sphere model in the simulation, where the particle size is denoted by Dk. The physical properties of these particles are outlined in Table 3.

**Table 3:** Pellet shape and material parameters.

Parameters	Value
Radius of the particle, mm	0.1
Density of the material, kg∙m^3^	2.7 × 10^3^
Poisson's ratio of the material	0.33
Shear modulus of the material, MPa	6.89 × 10^3^

#### Contact model

**Figure 3:**
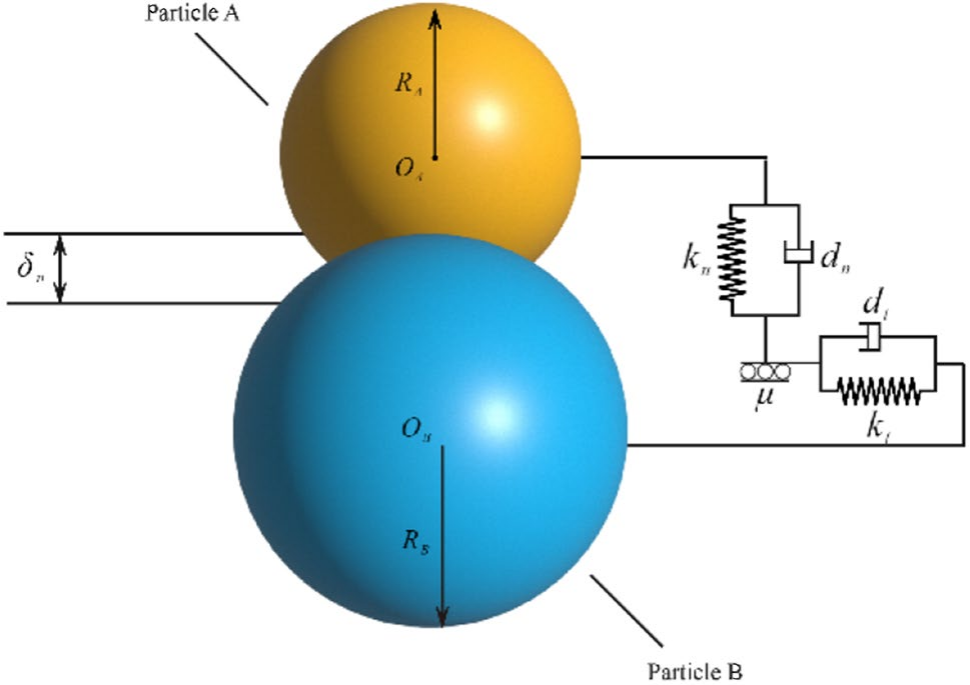
Schematic of the Hertz‒Mindlin no-slip contact model.

Since the introduction of the soft-sphere model, also known as the discrete cell method, this model has been widely employed in numerical simulations of various granular material dynamics, including particle separation, smelting, and impact dynamics^26–31^. There are primarily six contact models available, as indicated in Table 4. Scine no slip is being considered to occur between the powder particles, the widely used Hertz–Mindlin no-slip contact model^32,33^ is employed in the present study. This model is selected mainly because from of its the maturity and applicability .A schematic of this model is depicted in Fig. 3.

**Table 4:** Classification of the contact models.

Serial number	Contact model
1	Hertz‒Mindlin no-slip contact model
2	Hertz‒Mindlin adhesive contact model
3	Linear adhesion contact model
4	Moving Surface Contact Modeling
5	Linear elastic contact model
6	Frictionally charged contact model

In this model, the normal force between the particles, tangential force, normal damping force, and tangential damping force can be calculated using the following expressions:

7 $${F}_{n}=\frac{4}{3}{E}^{*}\sqrt{{R}^{*}{\alpha }^{3}}$$

8 $${F}_{t}=-{S}_{t}\delta $$

9 $${F}_{n}^{d}=-2\sqrt{\frac{5}{6}} \beta \sqrt{{S}_{n}{m}^{*}}{\upsilon }_{n}^{rel}$$

10 $${F}_{n}^{d}=-2\sqrt{\frac{5}{6}} \beta \sqrt{{S}_{n}{m}^{*}}{\upsilon }_{n}^{rel}$$

where ${E}^{*}$ is the equivalent modulus of elasticity, Pa; ${R}^{*}$ denotes the equivalent particle radius, m; $\alpha$ represents the normal overlap, m; $\delta$ is the tangential overlap, m; ${S}_{t}$ is the tangential stiffness, N/m; ${S}_{n}$ is the normal stiffness, N/m; ${m}^{*}$ represents the equivalent mass, kg; ${\upsilon }_{n}^{rel}$ denotes the normal relative velocity, m/s; and ${\upsilon }_{t}^{rel}$ represents the tangential relative velocity, m/s.

#### Simulation parameters

In this study EDEM software is used to perform the simulations. The simulation parameters are configured as outlined in Table 5. The coefficients of restitution, static friction, and rolling friction between the particles and the geometry were selected as to be 0.31, 0.61, and 0.47, respectively. These parameters are set based on the material of the rotor and the particles. Additionally, the coefficients between the particles were set to 0.1, 1.05, and 1.4.

**Table 5:** Simulation parameters.

Parameters	Value
Disk rotation speed, rpm	$1\times 1{0}^{4}$
Total number	$1\times 1{0}^{4}$
Target number	$2\times 1{0}^{6}$
Rayleigh time step	$2\times 1{0}^{-7}$
Simulation time, ms	55

#### Model boundary

In the high-speed internal flow of the rotor, the behavior of the particles becomes more complex. The overall speed change pattern of the particles is investigated in this study by setting different calculation domains. In this regard, the following three parameters are considered:

The average speed of particles thrown ${V}_{p}$: This parameter reflects the instantaneous speed of particles thrown out of the accelerator disc and is an index used to measure the acceleration ability. It can be calculated by collecting the average value of the particle speeds throughout the entire acceleration stage in Computational Domain A.

Average speed of the particles ${V}_{all}$: This parameter represents the average speed of all the particles during the entire acceleration process. It can be obtained by collecting the average value of all the particle velocities in Computational Domain B.

Particle velocity distribution *V*_*f*_ (t): This parameter illustrates the velocity distribution of particles at a specific moment (t = 30 ms) within Computational Domain B.

**Figure 4:**
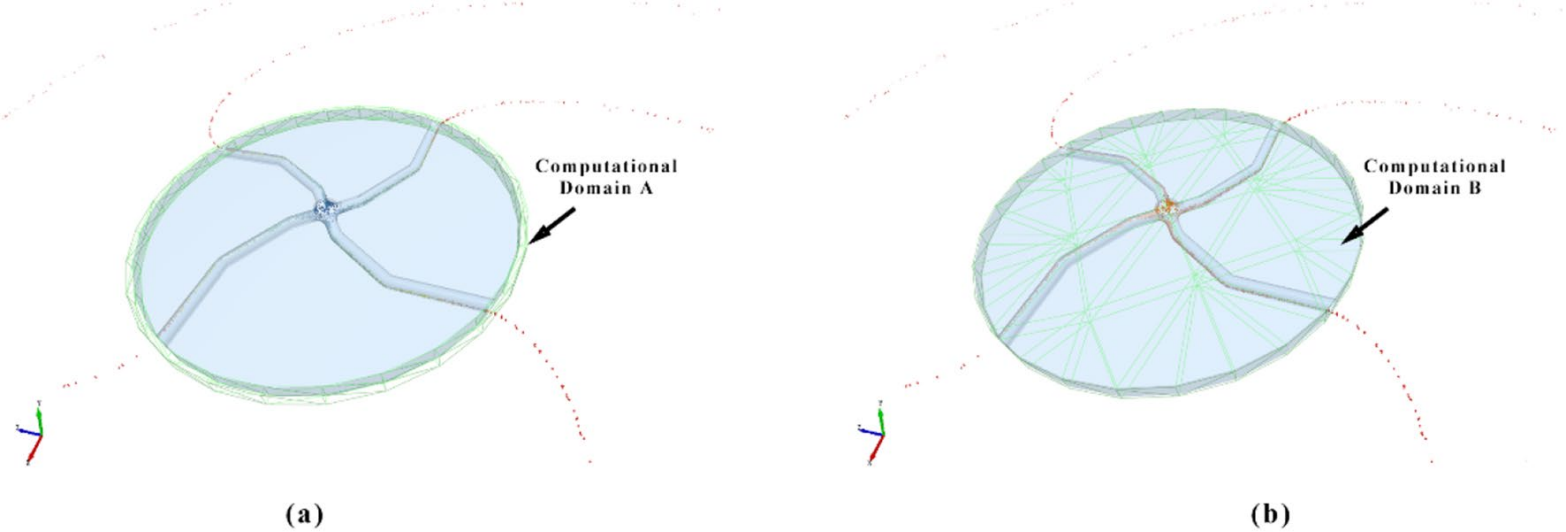
Computational domain.

The computational domains A and B are presented in Fig. 4.

### Limitations of the method

The objective of this study is to simulate the centrifugal motion process of metal particles using the discrete cell method. However, there are two limitations to this research process. First, the simulation environment is limited. To minimize the impact of gas flow on particle motion, the simulation of particle acceleration must be carried out in a vacuum, which simplifies the gas-particle two-phase flow in an actual experimental environment. Second, the experimental objects are limited. The materials of the centrifugal accelerating rotor and particles used in the study are restricted, and the differences in the static friction coefficient, sliding friction coefficient, and rolling friction coefficient among the different materials may affect the experimental results. Therefore, in future studies, we will introduce the gas-particle two-phase flow simulation method, and increase the types of rotor and particle materials to more accurately reflect the particle motion.

## Results and discussion

### Effect of different flow channel structures on the centrifugal acceleration of particles

#### Effect on the average speed of particles thrown ${V}_{p}$

**Figure 5:**
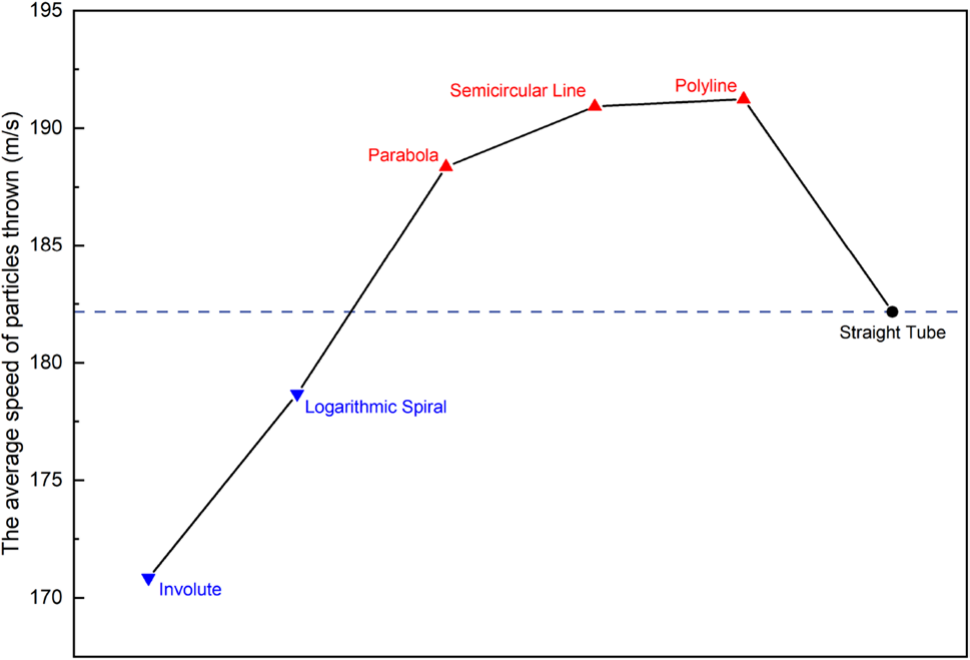
${V}_{p}$ of accelerator disks with different runner structures.

Figure 5 illustrates the average velocity of particles throw after being accelerated by different types of runner-type turntables. The through-hole type runner-accelerated turntable provides a benchmark with an average velocity of 182.16 m/s. The results for the parabolic, circular arc and multii-segmented line runner accelerated turntable are 188.36 m/s, 190.92 m/s, and 191.24 m/s, respectively. Notably, these values are higher than the benchmark values. On the other hand, the involute and logarithmic helix-type runner-accelerated turntables produced average velocities of 170.85 m/s and 178.68 m/s, respectively, which are lower than the benchmark values. This implies that under the same rotational speed and diameter conditions, the acceleration ability of turntables that contain parabolic, arc, and multii-segmented runners is superior to that of turntables using that contain involute and logarithmic helix runners.

#### Effect on the overall acceleration of the particles ${V}_{all}$

**Figure 6:**
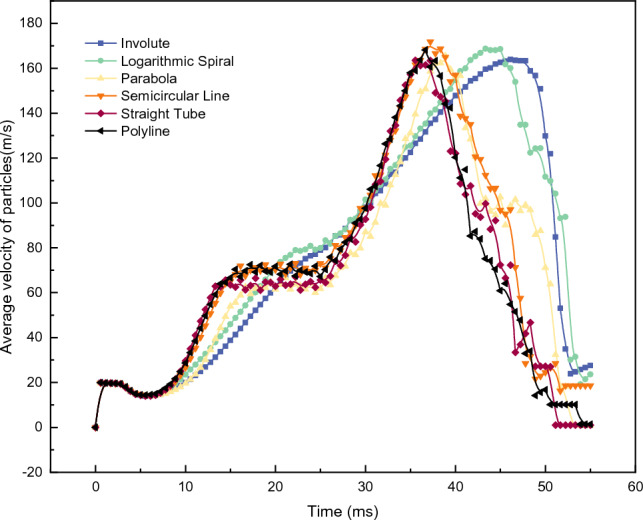
${V}_{all}$ of accelerator disks with different runner structures.

Figure 6 shows the change in the average velocity ${V}_{all}$ of all the particles in Computational Domain B over time. The overall trend exhibited five stages: slow decline, rise, plateau, rise, and rapid decline.

**Figure 7:**
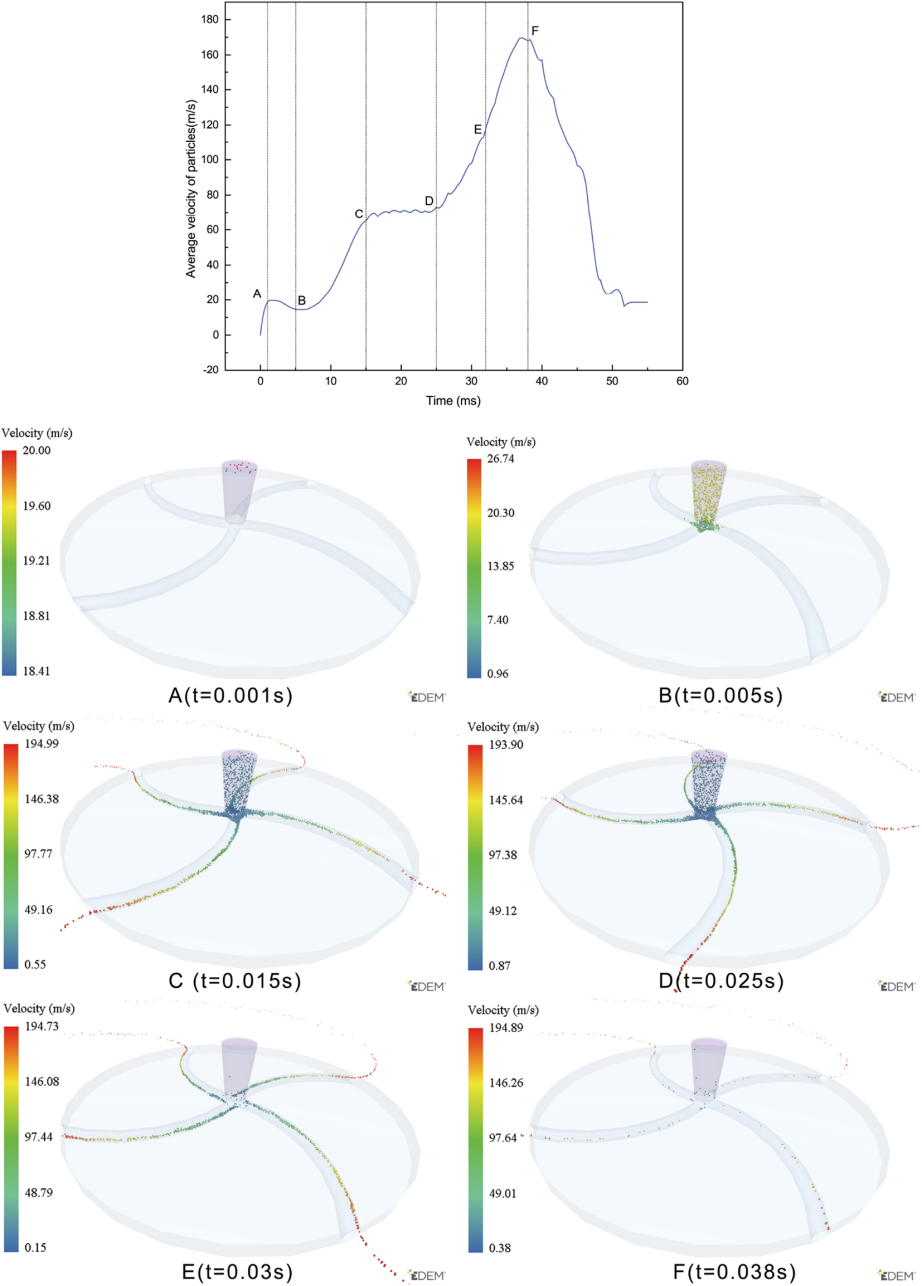
Accelerated state of the particles at different stages.

Taking the arc-type acceleration turntable as an example, the particle acceleration state corresponding to each stage is analyzed in Fig. 7. In the AB stage, the particles gradually fill the entire drop area and reach the turntable. The turntable starts to rotate at 10,000 rpm at Moment B, and the ${V}_{all}$ value decreases slightly during this stage. In the BC stage, particles are thrown out along the runner of the turntable due to the centrifugal effect, and particles flying away from the turntable begin to appear at Moment C. The ${V}_{all}$ value shows an upward trend in this stage. In Stage CD, there is a dynamic equilibrium between the number of newly generated unaccelerated particles and the number of accelerated particles that have separated from the turntable. The ${V}_{all}$ value remains at a plateau position until Moment D , when no new particles are generated. At this point, ${V}_{all}$ increases again. In Stage DF, all the particles are accelerated by the turntable. By this stage, all the particles have been accelerated and thrown out. The number of particles not accelerated in the disc is less than 1‰ at moment F, indicating that the accelerating process of the particles is essentially completed. The ${V}_{all}$ value reaches its peak at this moment and then declines rapidly. The analysis of each stage's particle state and the ${V}_{all}$ value reveals that the size of the ${V}_{all}$ value reflects different acceleration stages of the particles. The moment when ${V}_{all}$ reaches its peak value signifies the completion of particle acceleration. The changes in ${V}_{all}$, as shown in Fig. 6, indicate that the through-hole, parabolic, arc, and multii–segmented line-type accelerating carousels exhibit five clear stages. In contrast, the involute and logarithmic helix accelerating carousels have almost no plateau stage, indicating that the latter two types of carousels accelerate particles slowly, and a balance between the number of particles entering and leaving the carousel is never achieved. The time it takes for ${V}_{all}$ to reach its peak value also reflects a consistent pattern, with the involute and logarithmic spiral turntables lagging, indicating that it takes longer to accelerate all the particles to their maximum speed.

#### Effect on particle velocity distribution

**Figure 8:**
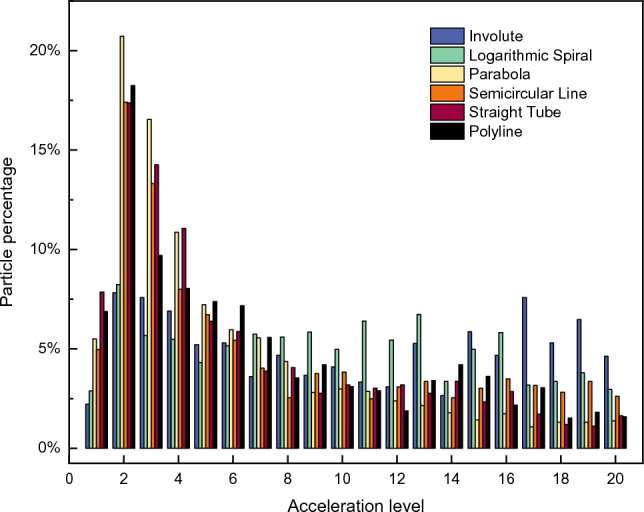
*V*_*f*_ (t) of the accelerator disks with different runner structures.

To investigate the uniformity of the particle acceleration process by different rotors, the distribution of particle velocities in Computational Domain B at t t = 30 ms was selected for study. The velocities of the particles in each rotor were divided into 20 classes, and the statistics of the number of particles in each class are shown in Fig. 8. The histogram distribution reveals that the velocities of the particles in the four types of accelerating rotors (parabolic, arc, through-hole, and polyline) are mainly concentrated in Grades 2–6. In contrast, the velocities of the particles in the two types of accelerating rotors (involute and logarithmic helices) are more uniformly distributed across the 20 grades. Further analysis of the percentage of particles in the 5 highest grades of the velocity class revealed that three types of rotors—involute, logarithmic helix, and circular arc—exceeded 10%, while the remaining three types of rotors did not. These results indicate that the accelerating rotors of the involute, logarithmic helix, and circular arc exhibit better uniformity in the overall acceleration process, and the velocity changes of the particles show better continuity in these cases.

The analysis demonstrates that the change in the runner structure has a significant impact on the centrifugal acceleration of the particles. Considering the average velocity of the particles ${V}_{p}$, the average velocity of the particles ${V}_{all}$, and the particle velocity distribution *V*_*f*_ (t), it is observed that the arc-shaped rotor provides optimal acceleration effects. Consequently, further investigation into the influence of runner structure parameters on the centrifugal acceleration of particles is conducted, specifically focusing on the arc-shaped accelerating rotor.

### Influence of different structural parameters of circular arc-linear flow channels on the centrifugal acceleration of particles

The parameters influencing the structural characteristics of the circular arc linear runner primarily include the curvature radius of the runner, the number of runners, and the cross-sectional dimensions of the runner. The aim of this study is to analyze the impact of each parameter change on the centrifugal acceleration effect of the particles via simulation of the effects of changing each parameter individually on the average throwing velocity ${V}_{p}$ and the overall average velocity of the particles ${V}_{all}$. The parameter settings for the simulation is outlined in Table 6.

**Table 6:** Parameter settings.

	Curvature radius mm	Number of runners	Cross-sectional dimensions mm
Curvature radius	80,100,120,160,320	4	10
Number of runners	120	2,3,4,5,6	10
Cross-sectional dimensions	120	4	7,8,9,10,11

#### Effect of different structural parameters on the average throw velocity ${V}_{p}$

**Figure 9:**
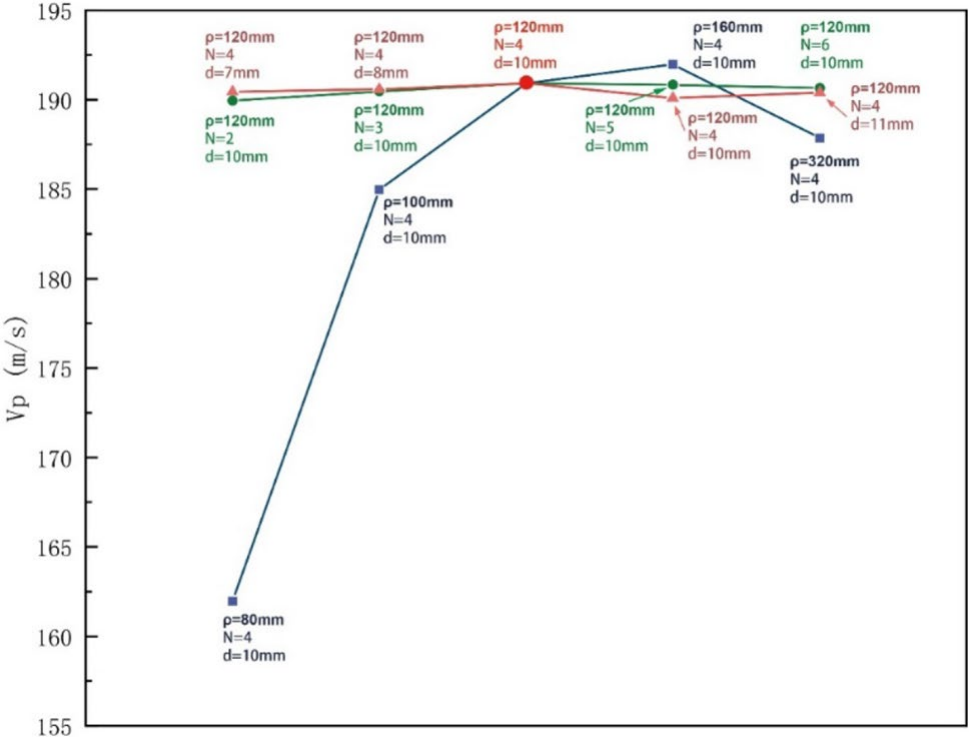
${V}_{p}$ of accelerator disks with different runner structural parameters.

Figure 9 illustrates the impact of various structural parameters of the rotor on the average throwing speed. The observed trend indicates that the curvature radius (*ρ*) of the runner has the most significant influence on the average throwing speed, while the number of runners (*N*) and the diameter of the runner cross-section (*d*) have almost no effect. Consequently, the analysis will primarily concentrate on examining the influence of the runner curvature radius on the average throwing speed.

The simulation was conducted with a rotor speed of 10,000 rpm, four flow channels, and an inner diameter of the flow channel cross-section set at 10 mm. The results of the simulation for different radii of curvature (*ρ*) of the rotor (80 mm, 100 mm, 120 mm, 160 mm, and 320 mm) revealed varying average particle throwing speeds (${V}_{p}$) of 161.97 m/s, 184.97 m/s, 190.92 m/s, 191.98 m/s, and 187.85 m/s, respectively. The trend in the values of ${V}_{p}$ indicates a sharp increase, slow increase, and slow decrease. Specifically, when the radius of curvature is 160 mm, the throwing speed reaches its peak. These findings reveal that for acircular curved-line type runner, a smaller radius of curvature results in a greater degree of curvature, making the runner's particle acceleration capability closer to that of involute-type and logarithmic spiral-type rotors. As the radius of curvature gradually increases, the degree of bend in the runner diminishes, the runner's form transitions towardds a straight-through type, and the acceleration ability of the particles decreases, becoming closer to it. According to reference 16, the angle of the guide plate has an impact on the direction of the relative velocity when particles are thrown. It has been found that the angle between the rotor radial direction and the particles can achieve maximum velocity at an inclination angle of 35°. This paper regulates the angle between the relative velocity and the radial direction of the rotor during particle throwing by adjusting the radius of curvature of the flow channel. The results obtained are consistent with the law reflected in the literature.

#### Effect of different structural parameters on the overall mean velocity of the particles ${V}_{all}$

**Figure 10:**
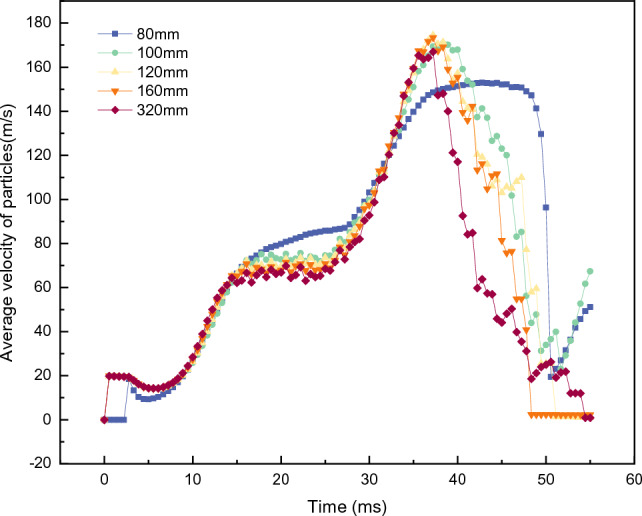
*V*_*all*_ of accelerator disks with different runner curvature radii.

Figure 10 illustrates the influence of the runner curvature radius on the overall average velocity of the particles ${V}_{all}$. The change in ${V}_{all}$ reveals that for a small curvature radius (*ρ* = 80 mm), the arc-line type acceleration rotor and the involute, logarithmic helix-type acceleration rotor have similar effects on the overall acceleration of the particles. The secondary increase in ${V}_{all}$ is not prominent enough, and a plateau appears in the ${V}_{all}$ peak area. This plateau corresponds to a state where more particles inside the rotor cannot be accelerated. As the radius of curvature gradually increases, ${V}_{all}$ shows obvious characteristics of a second increase,, and the points of rapid decrease are almost the same. This indicates that, with the gradual increase in the radius of curvature, the overall acceleration of the particles tends to be the same over time.

**Figure 11:**
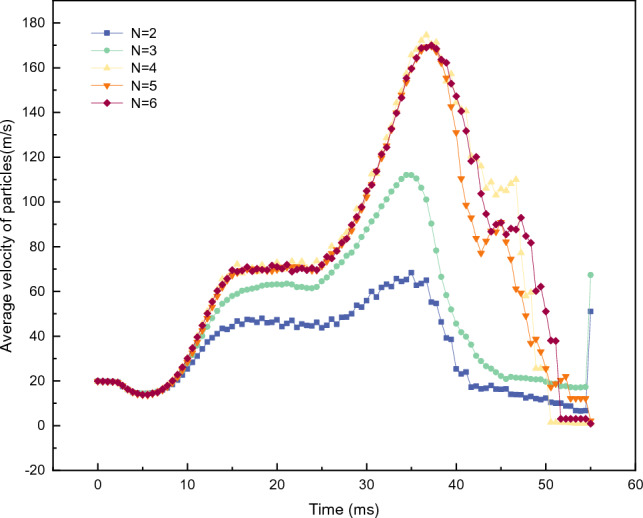
${V}_{all}$ of the accelerator disks with different numbers of runners.

Figure 11 illustrates the influence of different runner numbers on the overall average velocity of the particles. When *N* ≥ 4, the ${V}_{all}$ change curve tends to overlap, indicating that the change in the number of runners at this point has almost no effect on the acceleration of the particles. However, when *N* = 2 and 3, the peak value of ${V}_{all}$ declines significantly, and the duration of the decline phase increases. At this time, the situation of runner blockage inside the turntable is more obvious, indicating that the reduction in the number of runners not only decreases the acceleration of particles by the turntable but also weakens the ability of particles to be thrown out. The findings of reference 15 are consistent with the results obtained, indicating that the rotor structure of a vertical shaft impact crusher with six channels is the most reasonable and provides the best acceleration effect. However, a rotor with too many channels can cause most particles to leave the rotor without achieving full acceleration. On the other hand, fewer rotor channels will result in fewer particles thrown off the rotor per unit time, resulting in a longer acceleration time.

**Figure 12:**
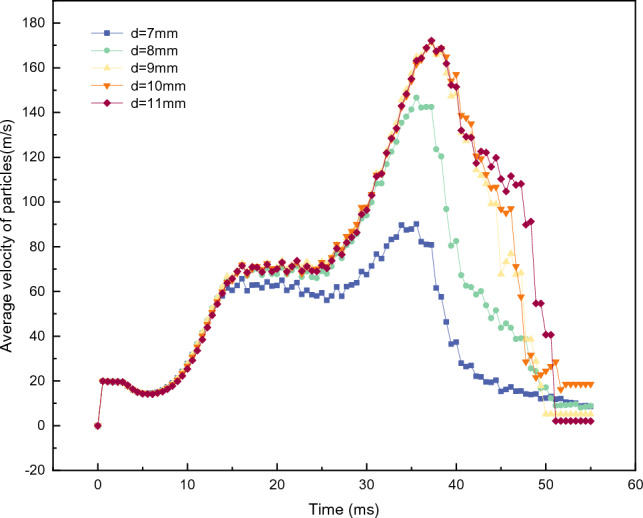
${V}_{all}$ of accelerator disks with different runner section diameters.

Figure 12 shows the effect of the runner cross-sectionsectional (*d*) on the overall average velocity (${V}_{all}$) of the particles. This indicates that the magnitude of *d* directly affects the passage performance of the runner. It is also observed that when *d* = 7 mm and 8 mm, the ${V}_{all}$ curve exhibits a lower peak value and a more gently descending phase curve. When *d* is 9 mm, 10 mm, and 11 mm, the curve trend becomes consistent, indicating that for 1 mm particle size, the ross-sectionsectional diameter of the flow channel should be at least 9 mm to ensure that the overall acceleration of the particles is not affected.

**Figure 13:**
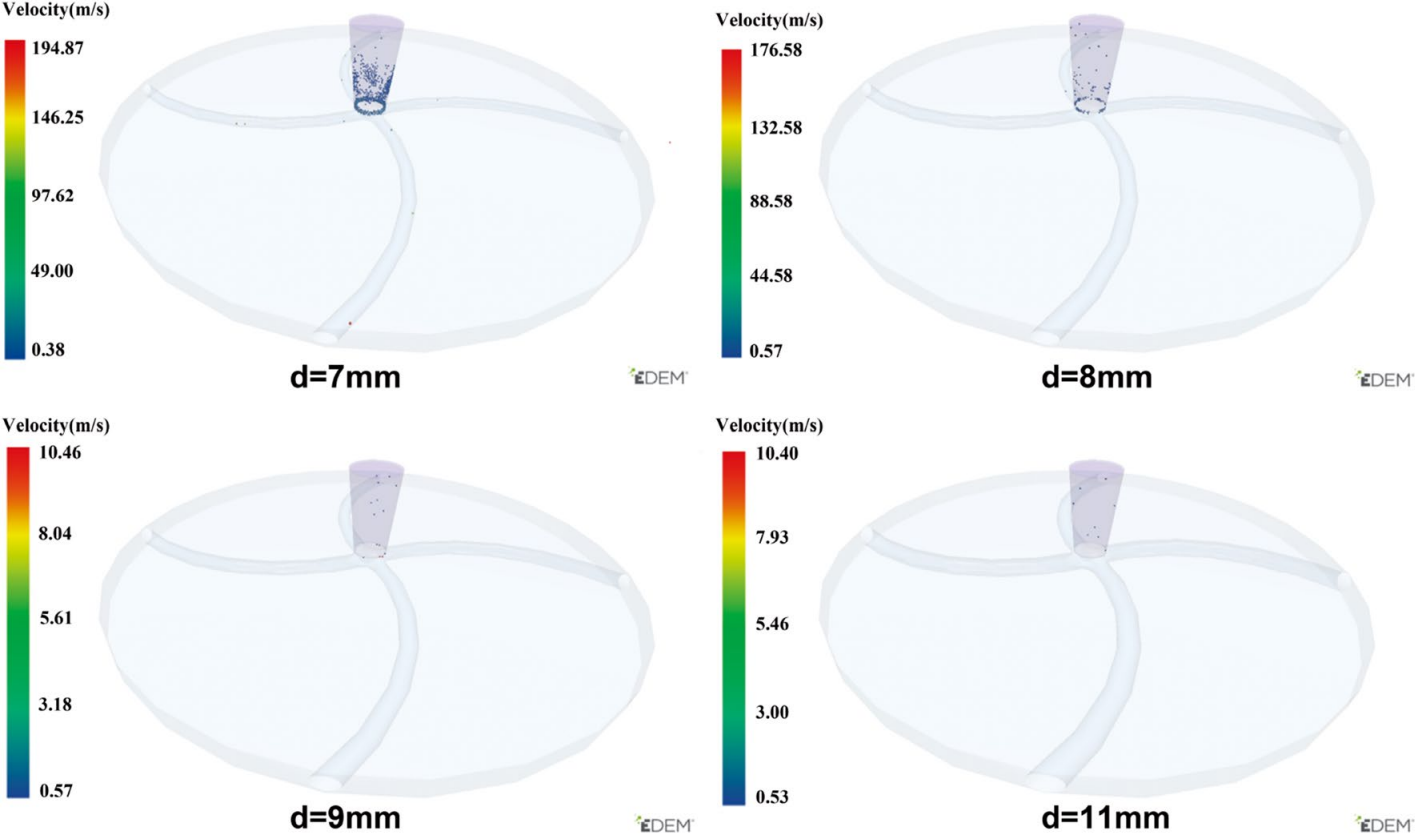
The particle state at the time of simulation termination in acceleration disks with different cross-sectional diameters of the runner.

Figure 13 shows the particles inside the rotor at the end of the simulation (t = 55 ms) when the cross-sectional diameter *d* is 7 mm, 8 mm, 9 mm and 11 mm. When *d* = 7 mm, 8 mm there is a larger number of particles remain inside the rotor, which further proves that the rotor cannot accelerate the particles and throw them out in the specified time under the two cross-sectional diameters and that the particles inside the rotor are prone to be blockage.

## Conclusion

The present study focuses on how six distinct runner structures influence the centrifugal acceleration of particles. The main achievements of this article can be summarized as follows:

1. This paper proposes three indicators for evaluating the acceleration performance of a centrifugal acceleration rotor. The average throwing speed ${V}_{p}$ and the average speed of particles ${V}_{all}$ reflect the ejection velocity achievable by particles in the rotor, while the analysis of variations of particle velocity distribution *V*_*f*_ (t) reflects the uniformity of the acceleration process. By analyzing the above indicators of centrifugal acceleration rotors with six different types of flow channels: involute, logarithmic spiral, parabolic, circular arc, straight through hole, and multi-segment line types, it is found that the circular arc flow channel acceleration rotor can achieve higher ejection velocity and better acceleration uniformity.
2. For the circular arc flow channel acceleration rotor, an analysis of the structural parameters on the particle acceleration process was conducted. The curvature radius *ρ* has a significant impact on the process of particle acceleration. The closer *ρ* is to the radius of the acceleration rotor, the greater ${V}_{p}$ that the particles can attain. The number of flow channels and the Cross-sectional dimensions have little effect on the particle ejection velocity ${V}_{p}$, but they do affect the particle pass-through rate of the flow channel. This pass-through rate increases with the increase of these two parameters, and decreases with their decrease. In particular, when the Cross-sectional dimensions are less than the boundary value, a large number of particles may not be ejected.
3. In future research, attention will be directed towards three primary areas. Firstly, the integration of the gas-particle two-phase flow simulation method will be explored to develop a particle acceleration model that incorporates particle kinetics and continuum mechanics. This endeavor aims to enhance the alignment of the particle acceleration process with real-world experimental conditions. Secondly, an expanded array of material categories for the centrifugal accelerating rotor and particles will be investigated to delve deeper into the impact of material properties on particle centrifugal acceleration. Lastly, experiments will be conducted employing pertinent equipment, and the ensuing results will be juxtaposed against simulation outcomes to iteratively refine the particle acceleration model.

## Data Availability

The datasets generated during and/or analyz during the current study are available from the corresponding author upon reasonable request.
